# Has the Lecture a Future in Education?

**Published:** 1905-08

**Authors:** 


### Editorial.

### HAS THE LECTURE A FUTURE IN EDUCATION?

Lectures have been from the remote periods of human history the favorite method of imparting information to the aspiring student and in many branches of professional work they have been, until recent years, the principal means of instruction.

The evolution of thought and the gradual unfolding of practical ideas has brought about a sceptical condition of mind in regard to this as a means of successful training, and there has grown in many quarters a revolt against what is regarded as a mediæval method of education, and thought to be unworthy this progressive and practical age.

A writer in the *British Dental Journal* of April 16, N. G. Bennett, ventures upon a philippic against the old practice of imparting information, and styles it the "Comedy of Lectures." Another writer in the present number, W. H. Trueman, quotes, with approbation, the action of Princeton in increasing the number of preceptors to keep in constant touch with students as "guides, advisors, and testers of their learning." The cry is by no means a new one. It has been sounded in the halls of professsional learning these many years, without apparently producing much effect. Sixty-six years ago (1839) Dr. Harris, in organizing the first dental college of the world, when he was refused co-operation by the medical educators of his city, made the first great innovation in professional teaching, and for this he has never received proper credit. He evidently was impressed with the futility of the course of lectures, as a sole means of educating the medical man, prevalent in his day, and in establishing his dental college he started out with the fixed idea that the student must be trained mainly upon the living subject. From that day to the present the dental student expects to secure his practical knowledge in the operative and prosthetic branches through this method. It is not at all probable that Harris appreciated the full extent and far-reaching possibilities of his wise procedure, and could he now witness the almost daily exhibit, in our large dental colleges, of probably a hundred and fifty men working over that many chairs and on patients under personal supervision, and performing delicate operations certain to be required in future practice, he would feel that this was by no means the least important work he did for his profession and for the improvement of general medical instruction.

The method that Harris inaugurated naturally led to an examination of the technical possibilities in teaching, and observant educators were not slow to see the fallacy of depending solely on didactic work. In the medical schools the old lecture system still held, partly through the force of tradition, and more from lack of means and room to establish personal supervision in laboratories adapted for the purpose. It is not many years since the new order came gradually into force, and the importance of the lecture has been greatly lessened. Men, not very old, can remember when chemistry was taught in lectures, when bacteriology was an unknown subject, and histology was regarded very much as a toy to while away an hour among the unknown mysteries of life. Now the graduate in dentistry and medicine would be deprived of a large portion of his professional skill were he suddenly to be bereft of the knowledge obtained in the laboratories devoted to these several subjects.

From working on the living subject to that devoted to technical training seems very much like reversing the natural order, but it was through observing the defects of the method of teaching in dental schools that led the observant mind to regard manual training as a necessary prerequisite, and the technical class-work was the natural result. It is some forty years since the mind of the present writer was forcibly awakened to the necessity of a change in the then existing methods of teaching dentistry. The time then was not ripe for radical measures in this direction, but in an experimental way a section upon histological studies was formed under his personal supervision. The success of this brought about, in after years, in the school with which he was connected, the technic training, and this has spontaneously become an essential part of the curricula of all the dental colleges of this country, and which has increased so much the development of dentistry in this part of the world.

While the value of this practical training cannot be overestimated, it must not be forgotten that the older methods have not, by any means, became a "comedy" to rouse the laugh of the unthinking. They are still the inspiring life of dental education and are full of meaning for the future.

Lectures are not to be relegated to the shelves of forgotten methods of teaching. They have performed a noble work in the past, and will continue to do it in the future. The part they must play will be to inspire the soul, that Haeckel says exists in all created things, and will make the dry body of mere technical teaching teem with life. The fault of this older method is not in the process, but in those who attempt to carry it out. To be a lecturer worthy the subject means three things,—first, to be able to think on the feet; second, to be thoroughly saturated with the subject; and third, to *never cease to train for every lecture.*

The series of lectures, as described by the English writer quoted, causes in the reading a sense of weariness of soul. It is true, as he writes, thus, "The systematic course of lectures on text-book subjects is, to a large extent, and for a number of men, a painful waste of time." Is that lecturing in the best sense? The man who places a manuscript between himself and his audience raises a barrier that genius may be unable to surmount. On the other hand, the man who stands before his class fully alive to the importance of his subject, inspired by practical experience, with text-books in the dark background, and only referred to, if at all, to enforce a point, notes confined to a few headings and rarely referred to,—such a lecture, delivered with earnestness and clear enunciation, will strike home as no amount of personal talk over the chair or in the laboratory can possibly effect. The philosophy of practice can be taught only in this manner. The life of the subject can be inspired here, and the class will be forced to think, to reason, and compare. The men never sleep under such a lecturer. They are imbued with the magnetic force of a great inspiration and such lectures live and become a vital energy long after their deliverance. Lectures have not had their day. They have come down to us through the centuries, and will continue a power in the centuries yet to be. How many of us can recall certain lectures of our student days that still continue with us as an inspiration! They have moulded our thoughts and made our lives more valuable. Why is it that Dr. Thomas Watson's lectures, delivered in London, on the "Principles and Practice of Physic," some sixty-nine years ago,—1836–37,—are to-day classic in medicine? It is only necessary to read these to secure the answer and, at the same time, to feel the inspiration that that model lecturer infused into them, and which will be felt as long as English medical literature exists.

There is too much of a disposition to consider old things as having lost their usefulness. The world cannot afford to part with anything that once possessed a certain value. It has taken centuries to mould into form the present methods of education, and our duty then is not to destroy but to build anew on this old foundation, combining past and present ideas, renewing the vitality of the present, leading directly to a broader superstructure that may develop, through experience, into better methods for the training of men.

